# Interactive Visualization Applications in Population Health and Health Services Research: Systematic Scoping Review

**DOI:** 10.2196/27534

**Published:** 2022-02-18

**Authors:** Jawad Chishtie, Iwona Anna Bielska, Aldo Barrera, Jean-Sebastien Marchand, Muhammad Imran, Syed Farhan Ali Tirmizi, Luke A Turcotte, Sarah Munce, John Shepherd, Arrani Senthinathan, Monica Cepoiu-Martin, Michael Irvine, Jessica Babineau, Sally Abudiab, Marko Bjelica, Christopher Collins, B Catharine Craven, Sara Guilcher, Tara Jeji, Parisa Naraei, Susan Jaglal

**Affiliations:** 1 Rehabilitation Sciences Institute Faculty of Medicine University of Toronto Toronto, ON Canada; 2 Toronto Rehabilitation Institute University Health Network Toronto, ON Canada; 3 Center for Health Informatics University of Calgary Calgary, AB Canada; 4 Alberta Health Services Edmonton, AB Canada; 5 McMaster University Hamilton, ON Canada; 6 Simon Fraser University Burnaby, BC Canada; 7 Universite de Sherbrooke Quebec, QC Canada; 8 Allama Iqbal Open University Islamabad Pakistan; 9 University of Waterloo Waterloo, ON Canada; 10 Department of Occupational Science and Occupational Therapy University of Toronto Toronto, ON Canada; 11 Institute of Health Policy, Management and Evaluation University of Toronto Toronto, ON Canada; 12 University of Calgary Calgary, AB Canada; 13 Department of Mathematics University of British Columbia Vancouver, BC Canada; 14 British Columbia Centre for Disease Control Vancouver, BC Canada; 15 Library & Information Services University Health Network Toronto, ON Canada; 16 The Institute for Education Research University Health Network Toronto, ON Canada; 17 Faculty of Science Ontario Tech University Oshawa, ON Canada; 18 KITE Toronto Rehabilitation Institute University Health Network Toronto, ON Canada; 19 Leslie Dan Faculty of Pharmacy University of Toronto Toronto, ON Canada; 20 Ontario Neurotrauma Foundation Toronto, ON Canada; 21 Department of Computer Science Ryerson University Toronto, ON Canada; 22 Department of Physical Therapy University of Toronto Toronto, ON Canada

**Keywords:** interactive visualization, data visualization, secondary health care data, public health informatics, population health, health services research

## Abstract

**Background:**

Simple visualizations in health research data, such as scatter plots, heat maps, and bar charts, typically present relationships between 2 variables. Interactive visualization methods allow for multiple related facets such as numerous risk factors to be studied simultaneously, leading to data insights through exploring trends and patterns from complex big health care data. The technique presents a powerful tool that can be used in combination with statistical analysis for knowledge discovery, hypothesis generation and testing, and decision support.

**Objective:**

The primary objective of this scoping review is to describe and summarize the evidence of interactive visualization applications, methods, and tools being used in population health and health services research (HSR) and their subdomains in the last 15 years, from January 1, 2005, to March 30, 2019. Our secondary objective is to describe the use cases, metrics, frameworks used, settings, target audience, goals, and co-design of applications.

**Methods:**

We adapted standard scoping review guidelines with a peer-reviewed search strategy: 2 independent researchers at each stage of screening and abstraction, with a third independent researcher to arbitrate conflicts and validate findings. A comprehensive abstraction platform was built to capture the data from diverse bodies of literature, primarily from the computer science and health care sectors. After screening 11,310 articles, we present findings from 56 applications from interrelated areas of population health and HSR, as well as their subdomains such as epidemiologic surveillance, health resource planning, access, and use and costs among diverse clinical and demographic populations.

**Results:**

In this companion review to our earlier systematic synthesis of the literature on visual analytics applications, we present findings in 6 major themes of interactive visualization applications developed for 8 major problem categories. We found a wide application of interactive visualization methods, the major ones being epidemiologic surveillance for infectious disease, resource planning, health service monitoring and quality, and studying medication use patterns. The data sources included mostly secondary administrative and electronic medical record data. In addition, at least two-thirds of the applications involved participatory co-design approaches while introducing a distinct category, *embedded research*, within co-design initiatives. These applications were in response to an identified need for data-driven insights into knowledge generation and decision support. We further discuss the opportunities stemming from the use of interactive visualization methods in studying global health; inequities, including social determinants of health; and other related areas. We also allude to the challenges in the uptake of these methods.

**Conclusions:**

Visualization in health has strong historical roots, with an upward trend in the use of these methods in population health and HSR. Such applications are being fast used by academic and health care agencies for knowledge discovery, hypotheses generation, and decision support.

**International Registered Report Identifier (IRRID):**

RR2-10.2196/14019

## Introduction

### Background

As digital medicine advances, visualization applications in population health increasingly provide ways for researchers and practitioners to explore and communicate findings [1], supporting knowledge discovery from disparate large data sources [2]. Visual analytics (VA) has been defined as the “science of analytics reasoning facilitated by visual interfaces” [3], and it is an interdisciplinary field combining visualization, statistical analysis, and advanced analytics such as machine learning and cognitive sciences [4]. A specific approach within VA is the use of interactive visualization, which Ola and Sedig [2] define as computational tools that store, process, and visually represent data, to facilitate interactive exploration. Interactive visualization increases the potential for big data use in health care by supporting sense making, knowledge discovery, and hypothesis generation [2,5]. Simple visualizations such as scatter plots, heat maps, and bar charts typically present 2 facets of the data, displaying attributes and relationships between 2 variables such as a disease condition and risk factors. Interactive visualization methods allow for presentation of multiple related facets such as risk factors to be studied simultaneously, leading to insights through exploring trends and patterns [2,5].

Population health research involves the study of data related to health outcomes and determinants of population health [6,7], whereas health services research (HSR) studies the health system in relation to access, quality, costs, and patient outcomes [8,9]. Both fields involve the analysis of large secondary data sources such as clinical databases, administrative data sets, and electronic medical records (EMRs) [10-12]. In a prior review, we summarized evidence on VA applications in these interrelated fields of health care [13]; this review complements it by reviewing the evidence on interactive visualization applications in population health and HSR.

Recent systematic reviews have summarized visualization methods in varied areas of health care. Among the most cited reviews, the study by West et al [1] synthesized literature on the use of visualization approaches for exploratory analysis of electronic health records (EHRs). Similarly, another well-cited review by Carrol et al [14] summarized the literature on visualization and analytics tools used in infectious disease epidemiology, particularly in relation to geographic information systems (GIS), molecular epidemiology, and social network analysis methods. Islam et al [15] offered a comprehensive view on data mining and theoretical approaches in health care. Wu et al [16] summarized evidence on visualization and analytic technologies for characterizing evaluation methods in health informatics, an area primarily concerned with clinical care. The most recent related review by Chung et al [17] focused on visual approaches in mental health care policy and systems. To our knowledge, interactive visualization applications have not been studied as a body of literature separate from data visualization and VA; hence, this review is the first systematic synthesis on the subject.

### Rationale for a Companion Review

This companion review is our second synthesis of literature on visualization and analytics tools, techniques, and approaches in population health and HSR. Our first publication focused on VA methods in these areas, where we offered an updated definition of VA in health care as “an approach, method, or application for analytic reasoning, exploration, knowledge discovery, and sense making of complex data, using one or more interactive visual interfaces, employing analytic and visual engines” [13]. As part of VA applications, analytic engines involve advanced machine learning, database querying, and manipulation.

*Interactive visualization* applications typically engage a front-end visual engine such as Tableau [18], Qlik [19], and PowerBI [20]. Although all VA methods carry a visualization component, which may or may not be interactive, interactive visualization applications typically do not involve or report an analytic component. Hence, this companion review on interactive visualization applications illustrates the state of evidence in population health and HSR, focusing on contemporary methods, approaches, tools, and co-design from real-world use cases. This review will be helpful for health care researchers, practitioners, and decision-makers to understand and adopt visualization-based data analysis.

### Objectives

The primary objective of this scoping review is to describe and summarize the evidence on interactive visualization applications, methods, and tools being used in population health and HSR and their subdomains in the last 15 years, from January 1, 2005, to March 30, 2019. Our secondary objective is to describe the use cases, metrics, frameworks used, settings, target audience, goals, and co-design of applications.

## Methods

### Review Methodology and Protocol

*Scoping reviews* outline the size and scope of available literature and identify the quality and extent of research evidence [21]. We briefly describe the methodological processes relevant to the second part of the review in this section, whereas further details can be found in the published protocol [22]. We primarily followed the guidance provided by the Joanna Briggs Institute [23], as well as the framework for conducting scoping reviews described by Arksey and O’Malley [24], with improvements suggested by Levac et al [25] and Peters et al [26], while using the PRISMA-ScR (Preferred Reporting Items for Systematic Reviews and Meta-Analyses Extension for Scoping Reviews) checklist provided by Tricco et al [27] for reporting. The major steps were as follows: determining the research question, identifying relevant studies, abstracting data, and summarizing and reporting the results. The operational concepts and definitions are presented in Textbox 1.

**Operational concepts and definitions.**

**Concepts and definitions**

- Population health, adapted from Kindig and Stoddart [6] and Kindig [7]
  - “The health outcomes of a group of individuals, including the distribution of such outcomes within the group,” includes “health outcomes, patterns of health determinants, and policies and interventions that link these two”
- Health services research, adapted from the Canadian Institutes of Health Research [8] and National Libraries of Medicine filters for health services research [28]
  - Research with the “goal of improving the efficiency and effectiveness of health professionals and the health care system”
  - Access to services
  - Utilization of services
  - Cost of services
- Domains of population health and health services research, adapted from Islam et al [15]
  - Clinical populations include a health condition
  - Epidemiologic includes disease distribution and dynamics
  - Demographic includes population-related characteristics such as age and gender
  - Spatiotemporal includes events over time and space
- Problem categories, based on subject area and the aim or aims of the application
  - Epidemiologic monitoring or surveillance
  - Resources and services monitoring and planning
  - Medication use patterns
  - Visualization methodologies
  - Epidemiologic data exploration
  - Health service monitoring, planning, and quality
  - Patient or care pathways
  - Public or patient communication
- Interactivity, adapted from Ola and Sedig [29] and Pike et al [30]
  - Ability to reflect changes in the visual representation, based on one or more variables available on the analytic interface
  - Tasks such as filtering, determining ranges, and finding anomalies, clusters, and the like by providing menus, dropdowns, and other options on the visualization interface
- Tools
  - Software for developing an application
- Use case
  - Use of the application or method to one or more data sets
- Goal of the application, adapted from Islam et al [15]
  - Whether the application was meant for decision support, knowledge discovery, or both
- Analytic capability, adapted from Islam et al [15]
  - Descriptive or predictive analytics or visual exploration of data
- Functions of the visualization presentations from the Graphic Continuum by Schwabish and Ribecca [31]
  - Spatial
  - Change over time
  - Flow
  - Distribution
  - Ranking
  - Magnitude
  - Correlation
  - Part to whole
- Co-design, adapted from Ward et al [32]
  - Encompasses the partnership of health workers, patients, and designers who aspire toward change, depending on shared knowledge to achieve “better outcomes or improved efficiency”
  - Whether any participatory approach toward co-design was reported by the authors
  - Embedded research: applications developed in response to an expressed need within a health care organization
- Settings and target audience
  - On the basis of the location of the application developed and the overall objectives of the reported application
  - Categories include academia, government health care units, and industry
- Subject of applications
  - Exploratory word frequency analysis of included articles to yield major subject areas for which applications were developed or any other related finding using a word cloud
- Applications in current use, public availability, innovation, and limitations
  - For ascertaining whether the application could be adapted or replicated in future
  - Public availability to ascertain whether the application was developed for the public

### Eligibility Criteria

Eligible articles included peer-reviewed published journal and full conference papers in English related to use cases of interactive visualization in population health and HSR. We included articles on spatiotemporal visualization but excluded articles presenting cartographic methods and tools for GIS because these were outside the scope of the research objectives. Similarly, we did not include articles on human-computer interaction, user design, and articles without a use case. Non–peer-reviewed work such as editorials, conference abstracts, and short articles were excluded. The eligibility criteria are presented in Textboxes 2 and 3.

**Inclusion criteria.**

**Inclusion criteria**

- Peer reviewed journal or full conference papers
- From January 1, 2005, to March 30, 2019
- Population health or health services research related
  - Articles with population level or health services research metrics: incidence, prevalence, events over time, and space, access, cost, utilization, disease or condition distribution, as well as social or multiple determinants of health
- Interactive visualization used for a use case with one or more data sets

**Exclusion criteria.**

**Exclusion criteria**

- Articles not in English
- Editorials, projects, reviews, book chapters, short papers, or reports
- Articles on computer vision and medical imaging
- Studies conducted in clinical settings without a population level or health services component, such as from a single hospital or unit
- Articles on device or sensor data, without a population level or health services research component
- Studies reporting a visual analytics component or analytic engine

### Sources of Evidence and Search Strategy

The search strategy, its conceptualization, and steps for operationalization are detailed in the review protocol [22]. The search was externally peer reviewed using the Peer Review of Electronic Search Strategies Guideline [33] and included an extensive list of search terms and their variants to cover all related concepts of population health, HSR, visualization, analytics, and interactivity [22]. The 6 databases searched, their platforms, and results are summarized in Table 1. We further hand searched 10 relevant journals, in addition to internet searches [22]. We used the Covidence (Veritas Health Innovation Ltd) platform for screening citations [34] and EndNote (Clarivate) for reference management [35].

**Table 1:** Databases and search results (N=14,099).

Database name	Search results, n (%)
MEDLINE (life sciences and biomedicine)	4633 (32.86)
Embase (life sciences and biomedicine)	1880 (13.33)
Web of Science (multidisciplinary)	5396 (38.27)
Ei Compendex (engineering and technology)	1267 (8.99)
IEEE Xplore (engineering and technology)	151 (1.07)
Inspec (engineering and technology)	772 (5.48)

### Data Charting and Synthesis of Results

In all, 2 independent reviewers screened articles at each stage of the review, including title and abstract screening, full-text screening, and data abstraction. A third reviewer acted as an arbiter in case of conflicts and for validating the data abstracted for their content and level of detail.

The data abstraction encompassed the major concepts in 6 major themes: (1) study characteristics (country, problem category, settings, and target audience), (2) tools and techniques used, (3) data type and visualization methods, (4) domains of population health and HSR, (5) innovation of the application and its current availability and use, and (6) if the application was co-designed with the target audience.

## Results

### Selection of Sources and Presentation of Results

**Figure 1:**
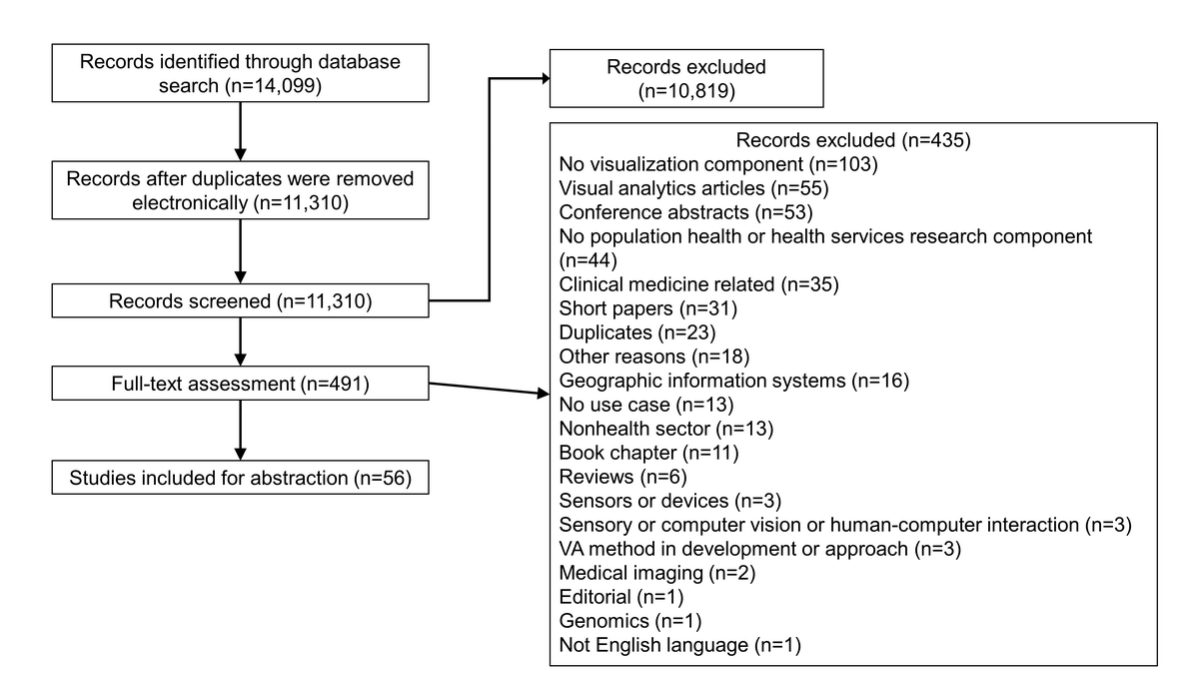
PRISMA (Preferred Reporting Items for Systematic Reviews and Meta-Analyses) flowchart for article selection. VA: visual analytics.

We identified 14,099 articles from the 6 databases searched. Given the varied sources of the articles, we adapted the method described by Bramer et al [36] for removing duplicate references using EndNote X9 [35]. Among the 14,099 articles, we considered major citation details and identified, double-checked, and removed 2078 (14.74%) duplicates, comparing the title, identifiers, publication platforms, and abstracts. From the remaining 12,021 articles, another 711 (5.91%) duplicates were removed after importing into Covidence [34]. We excluded 96% (10,819/11,310) of the references during the title and abstract screening and 89% (435/491) of the articles during the full-text screening. We did not find additional articles from reference lists of recent systematic and narrative reviews, hand searches of individual journals, and internet searches. Hence, of the initially identified 14,099 articles, we have summarized 56 (0.39%) for reporting in this review. The reasons for exclusion during the full-text-screening are detailed in the PRISMA (Preferred Reporting Items for Systematic Reviews and Meta-Analyses) diagram (Figure 1), whereas the Preferred Reporting Items for Systematic Reviews and Meta-Analyses Extension for Scoping Reviews reporting checklist is presented in Multimedia Appendix 1.

**Figure 2:**
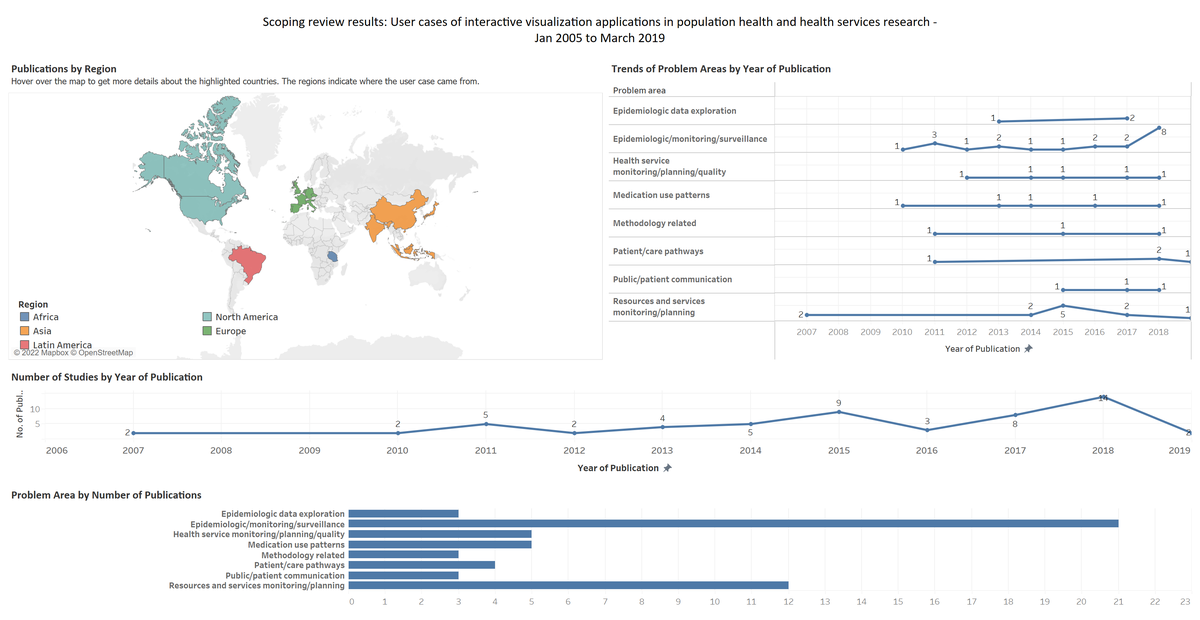
Screenshot of the results presented as a Tableau dashboard.

We have also summarized our results in a visual format using a publicly accessible Tableau dashboard, a screenshot of which is presented in Figure 2 [37]. The abstracted data and complete workbook are available to support replication, adaptation, and further analysis. Operational concepts for each category and reported theme are detailed in the *Methods* section (Textbox 1).

### Study Characteristics, Settings, and Target Audience

The 56 articles summarized were from 21 countries, including the United States (30/56, 54%), the United Kingdom (4/56, 7%), India (2/56, 4%), Indonesia (2/56, 4%), and Canada (2/56, 4%). Of the 56 articles, there was 1 (2%) each from the Netherlands, Spain, Puerto Rico, Czech Republic, Malaysia, France, Portugal, Tanzania, Slovenia, China, Germany, Brazil, Italy, Japan, and Korea, whereas 1 (2%) study included a comparison of health indicators from the United States, the United Kingdom, Costa Rica, Sweden, Croatia, Japan, Hong Kong, and China. Details on countries, settings, and target audiences are presented in Multimedia Appendix 2 [38-93], whereas these are summarized in Tables 2 and 3. Study settings included government ministry or health unit (39/56, 70%), academia (18/56, 32%), and industry (2/56, 4%). There was overlap between the government health unit and academia (1/56, 2%) and between the government health unit and industry (2/56, 4%).

The included studies often had more than one target audience. These were population or public health practitioners (53/56, 95%), clinicians (24/56, 43%), policy makers and decision-makers (21/56, 38%), public and patient groups (12/56, 21%), data scientists (5/56, 9%), and industry (2/56, 4%).

**Table 2:** Settings of the studies (N=56).

Setting	Values, n (%)	Study
Government; ministry; health department	39 (70)	Alibrahim et al (2014) [38], Barrento and De Castro Neto (2017) [39], Basole et al (2015) [40], BenRamadan et al (2017) [43], BenRamadan et al (2018) [44], Bjarnadottir et al (2016) [46], Brownstein et al (2010) [47], Henley et al (2018) [52], Hosseinpoor et al (2018) [53], Jia et al (2015) [56], Kirtland et al (2014) [58], Ko and Chang (2018) [59], Kubasek et al (2013) [61], Lanzarone et al (2016) [62], Lopez-DeFede et al (2011) [63], Mahler et al (2015) [64], Marshall et al (2017) [65], Mitrpanont et al (2017) [67], Moni et al (2015) [68], Monsen et al (2015) [69], Monsivais et al (2018) [70], Mozumder et al (2018) [71], Pachauri et al (2014)[73], Palmer et al (2019) [74], Pike et al (2017) [76], Podgornik et al (2007) [77], Pur et al (2007) [78], Raghupathi and Raghupathi (2018) [79], Ratwani and Fong (2015) [80], Rodriguez-Fernandez et al (2016) [81], Rowlingson et al (2013) [82], Shen et al (2018) [84], Sims et al (2011) [85], Sopan et al (2012) [86], Toyoda and Niki (2015) [87], Valdiserri and Sullivan (2018) [89], van der Corput et al (2014) [90], Wang and Yao (2018) [92], and Zhang et al (2011) [93]
Academia	18 (32)	Becnel et al (2019) [41], Benítez et al (2017) [42], Bieh-Zimmert et al (2013) [45], Bjarnadottir et al (2016) [46], Cesario et al (2012) [48], Chui et al (2011) [49], Haque et al (2014) [50], Happe and Drezen (2018) [51], Hsu et al (2018) [54], Iyer et al (2017) [55], Kaushal et al (2018) [57], Krause (2015) [60], Martinez et al (2016) [66], Ortiz-Zuazaga et al (2015) [72], Pickle and Carr (2010) [75], Semple et al (2013) [83], Tsoi et al (2018) [88], and Wang et al (2011) [91]
Industry	2 (4)	Ratwani and Fong (2015) [80] and Shen et al (2018) [84]

**Table 3:** Target audience of the included studies (N=56).

Target audience	Values, n (%)	Study
Population or public health practitioners	53 (95)	Alibrahim et al (2014) [38], Barrento and De Castro Neto (2017) [39], Becnel et al (2019) [41], Benitez et al (2017) [42], BenRamadan et al (2017) [43], BenRamadan et al (2018) [44], Bieh-Zimmert et al (2013) [45], Bjarnadottir et al (2016) [46], Brownstein et al (2010) [47], Cesario et al (2012) [48], Chui et al (2011) [49], Haque et al (2014) [50], Happe and Drezen (2018) [51], Henley et al (2018) [52], Hosseinpoor et al (2018) [53], Hsu et al (2018) [54], Iyer et al (2017) [55], Jia et al (2015) [56], Kaushal et al (2018) [57], Kirtland [58] 2014, Krause (2015) [60], Kubasek et al (2013) [61] , Lopez-DeFede et al (2011) [63], Mahler et al (2015) [64], Marshall et al (2017) [65], Martinez et al (2016) [66], Mitrpanont et al (2017) [67], Moni et al (2015) [68], Monsen et al (2015) [69], Monsivais et al (2018) [70], Mozumder et al (2018) [71], Ortiz-Zuazaga et al (2015) [72], Pachauri et al (2014) [73], Palmer et al (2019) [74], Pickle and Carr (2010) [75], Pike et al (2017) [76], Podgornik et al (2007) [77], Pur et al (2007) [78], Raghupathi and Raghupathi (2018) [79], Ratwani and Fong (2015) [80], Rodriguez-Fernandez et al (2016) [81], Rowlingson et al (2013) [82], Semple et al (2013) [83], Shen et al (2018) [84], Sims et al (2011) [85], Sopan et al (2012) [86], Toyoda and Niki (2015) [87], Tsoi et al (2018) [88], Valdiserri and Sullivan (2018) [89], van der Corput et al (2014) [90], Wang et al (2011) [91], Wang and Yao (2018) [92], and Zhang et al (2011) [93]
Clinicians	24 (43)	Basole et al (2015) [40], Becnel et al (2019) [41], BenRamadan et al (2017) [43], BenRamadan et al (2018) [44], Bjarnadottir et al (2016) [46], Brownstein et al (2010) [47], Haque et al (2014) [50], Happe and Drezen (2018) [51], Henley et al (2018) [52], Jia et al (2015) [56], Kaushal et al (2018) [57], Kirtland et al (2014) [58], Ko and Chang (2018) [59], Lanzarone et al (2016) [62], Marshall et al (2017) [65], Mitrpanont et al (2017) [67], Monsen et al (2015) [69], Mozumder et al (2018) [71], Palmer et al (2019) [74], Pike et al (2017) [76], Ratwani and Fong (2015) [80], Rodriguez-Fernandez et al (2016) [81], Semple et al (2013) [83], and van der Corput et al (2014) [90]
Policy makers and decision-makers	21 (38)	Alibrahim et al (2014) [38], Becnel et al (2019) [41], Hsu et al (2018) [54], Jia et al (2015) [56], Lanzarone et al (2016) [62], Mahler et al (2015) [64], Marshall et al (2017) [65], Moni et al (2015) [68], Monsen et al (2015) [69], Monsivais et al (2018) [70], Pike et al (2017) [76], Podgornik et al (2007) [77], Pur et al (2007) [78], Raghupathi and Raghupathi (2018) [79], Rowlingson et al (2013) [82], Semple et al (2013) [83], Sims et al (2011) [85], Sopan et al (2012) [86], Toyoda and Niki (2015) [87], Valdiserri and Sullivan (2018) [89], Wang (2018) [92], and Zhang et al (2011) [93]
Public and patient groups	12 (21)	Barrento and De Castro Neto (2017) [39], Bieh-Zimmert et al (2013) [45], Brownstein et al (2010) [47], Hosseinpoor et al (2018) [53], Hsu et al (2018) [54], Jia et al (2015) [56], Kubasek et al (2013) [61], Mozumder et al (2018) [71], Ortiz-Zuazaga et al (2015) [72], Semple et al (2013) [83], Tsoi et al (2018) [88], and van der Corput et al (2014) [90]
Data scientists	5 (9)	BenRamadan et al (2017) [43], Pickle and Carr (2010) [75], Tsoi et al (2018) [88], Valdiserri and Sullivan (2018) [89], and Wang et al (2011) [91]
Industry (software, pharmaceutical, and insurance)	2 (4)	Kaushal et al (2018) [57] and Toyoda and Niki (2015) [87]

### Health Care Domains, Metrics, and Categories of Problems Addressed by the Applications

Among the domains of health, the categories overlapped, with articles falling under population health (38/56, 68%), HSR (29/56, 52%), and both population health and HSR (11/56, 20%). Among the articles in the population health category, their subdomains included clinical populations with 1 condition of interest (23/56, 41%), demographic population (28/56, 50%), epidemic monitoring and modeling (11/56, 20%), and spatiotemporal (16/56, 29%). For HSR, these included access to services (16/56, 29%), utilization (23/56, 41%), and costs (4/56, 7%).

The visual applications for these health care areas used different metrics in combination with the major categories, including prevalence (23/56, 41%), space and time (20/56, 36%), incidence (19/56, 34%), resources (6/56, 11%), mortality (4/56, 7%), hospitalization (1/56, 2%), events over time (1/56, 2%), and air quality (1/56, 2%).

The problem categories addressed by the applications included epidemiologic monitoring or surveillance (21/56, 38%), resources and services monitoring or planning (12/56, 21%), health service monitoring or planning or quality (5/56, 9%), medication use patterns (5/56, 9%), patient or care pathways (4/56, 7%), visualization methodologies (3/56, 5%), epidemiologic data exploration (3/56, 5%), and public or patient communication (3/56, 5%).

### Application’s Analytic Capability, Goal, and Frameworks Used

There was overlap in the analytic capability of the tools with applications capable of descriptive analytics (53/56, 95%), predictive analytics (4/56, 7%), and visual exploration of complex data sets (37/56, 66%). Regarding the goal of the visualization application, there was overlap between knowledge discovery (56/56, 100%) and decision support (47/56, 84%). Of the 56 articles, 6 (11%) used a framework in their methods for developing the application. These frameworks are summarized in Table 4. Multimedia Appendix 3 [38-93] lists the analytic capability and goals of each application.

**Table 4:** Articles mentioning the use of methodological frameworks (N=6).

Author and year	Methodological frameworks used in developing interactive visualization applications
Alibrahim et al (2014) [38]	Display principles for visual monitoring by Few et al [94]
Bieh-Zimmert et al (2013) [45]	Ten guidelines by Kelleher and Wagener [95]
Monsen et al (2015) [69]	Followed the Omaha System [96]
Ratwani et al (2015) [80]	Visualization principles (overview, zoom and filter, and details on demand) based on theories from Shneiderman [97] and Chen [98]
Semple et al (2013) [83]	For developing the web app, the 5-stage user-centered design model described by Kinzie et al [99] was used
Wang et al (2011) [91]	Align, Rank, and Filter Framework used for user interaction by Wang et al [100]

### Data Characteristics: Source, Structure, Type, and Use Cases

Data sets used in the visualization applications were single (40/56, 71%) or multiple (16/56, 29%), and they were structured (48/56, 86%) or semistructured (8/56, 14%). The sources of data included administrative (45/56, 80%), spatiotemporal (17/56, 30%), EMR or EHR or medical records (15/56, 27%), registry (10/56, 18%), web or social media (2/56, 4%), and sensor data (1/56, 2%). Multimedia Appendix 4 [38-93] details the data types and sources with the primary tools used to develop the application.

### Visualization: Primary Types, Presentation, and Tools

Regarding the functional aspects of the interactive visual presentations, the categories included spatial (31/56, 55%), change over time (9/56, 16%), flow (8/56, 14%), distribution (2/56, 4%), ranking (2/56, 4%), magnitude (2/56, 4%), correlation (1/56, 2%), and part to whole (1/56, 2%).

**Figure 3:**
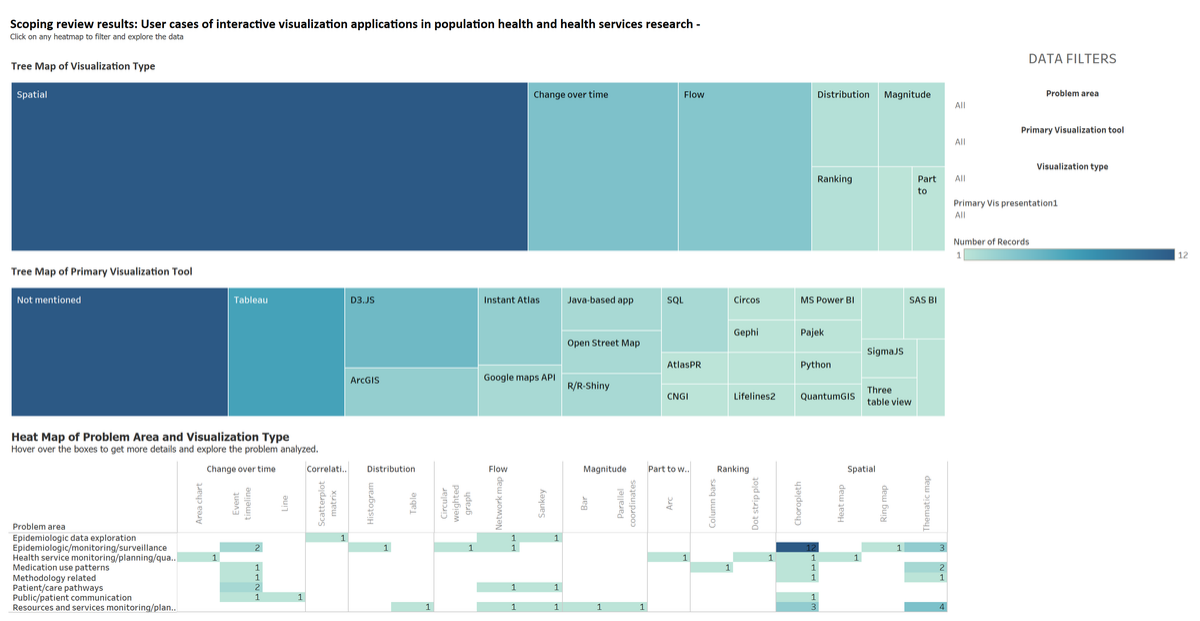
Types of visualizations, primary software tool, and visualization type by problem area (screenshot).

The primary visual presentations included choropleth map (19/56, 34%), thematic map (10/56, 18%), event timeline (7/56, 13%), network map (4/56, 7%), Sankey diagrams (3/56, 5%), area chart (1/56, 2%), parallel coordinates (1/56, 2%), column bars (1/56, 2%), circular weighted graph (1/56, 2%), line (1/56, 2%), dot strip plot (1/56, 2%), ring map (1/56, 2%), table (1/56, 2%), scatterplot matrix (1/56, 2%), bar (1/56, 2%), histogram (1/56, 2%), arc (1/56, 2%), and heat map (1/56, 2%). The relative distribution of visual presentations and software tools by problem category is provided in Figure 3. For details on the functional types and visual presentations included in each article, please refer to Multimedia Appendix 5 [38-93].

**Figure 4:**
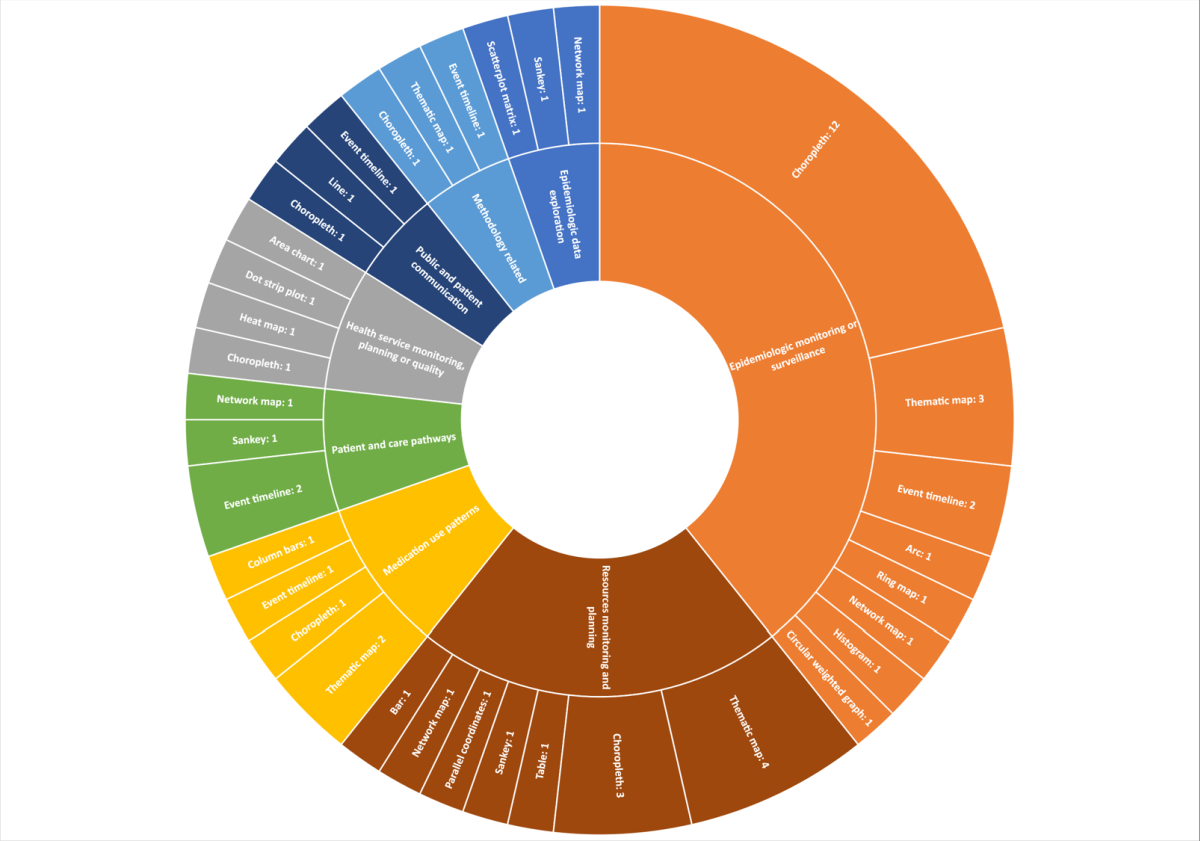
Primary visualization presentations by health care problem area.

The different visualization software tools used included Tableau (7/56, 13%); D3.JS (5/56, 9%); ArcGIS and Instant Atlas (3/56, 5% each); R/R-Shiny, Open Street Map, Google Maps application programming interface (API), SQL, and Java-based application (2/56, 4% each); and MS Power BI, SigmaJS, RESTful API, CNGI, Lifelines2, AtlasPR, Circos, IBM Watson Analytics, SAS BI, Pajek, Gephi, pChart, Three Table View, Python, and QuantumGIS (1/56, 2% each). Some articles did not mention the visualization tool (13/56, 23%). Figure 4 shows a screenshot from the Tableau results dashboard with the primary visualization tools and heat map of problem category and visual presentation. This interactive dashboard is also available on the Tableau results dashboard [37].

### Application Co-design

**Figure 5:**
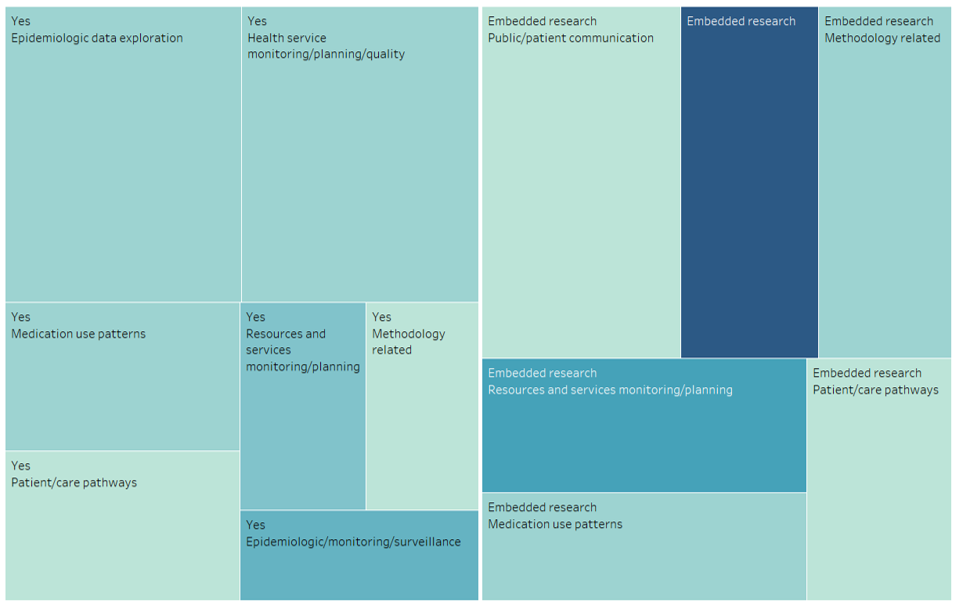
Co-designed applications and related health care areas (screenshot).

For participatory approaches to application development, some articles (35/56, 67%) mentioned co-design. Among these (20/35, 57%) were applications that were part of embedded research at health care organizations. Other articles (20/56, 37%) did not mention this aspect. Application co-design was found in the problem categories of epidemiologic monitoring or surveillance (13/56, 23%), resource and service monitoring and planning (8/56, 14%), medication use patterns (4/56, 7%), visualization methodology (3/56, 5%), epidemiologic data exploration (2/56, 4%), health service monitoring or planning or quality (2/56, 4%), patient or care pathways (2/56, 4%), and public or patient communication (1/56, 2%). Figure 5 shows a tree map of co-designed applications and embedded research.

### Applications in Current Use and Public Availability

Most of the applications were mentioned as being currently available and in use (31/56, 55%). Related to public access, a third of the applications were available to the public (18/56, 32%). There were applications using free or open source tools (18/56, 32%) and those using proprietary tools (19/56, 34%), or the tools were not mentioned (18/56, 32%).

## Discussion

### Significance of the Review

Data visualization in health has a lengthy history going back to the influential work of John Snow and Florence Nightingale in the 19th century. The field of interactive visualization has developed in parallel with computing power and the availability of large, complex health care data sets for diverse audiences such as clinicians, public health researchers, practitioners, and decision-makers [1,41], with considerable progress made in design methodologies [14]. Our review is a novel synthesis and summary of the literature from a vast body of research that had not previously been covered.

In this methodological review, we aim to capture the current state of knowledge and evidence on the topic of *interactive visualization applications* in population health and HSR, distinguishing them from conventional graphical presentations in health care and the related field of VA. We explored areas in population health and HSR to ascertain where these techniques have been used and identified trends and opportunities for the use of these applications.

As population health and health services researchers and practitioners, our perspective and interest in pursuing this research question were based on developing an in-depth understanding of the state of evidence on the use of visualization-based approaches for big health care data analyses. We anticipated that the review would help diverse audiences in population health and HSR learn from practical applications, inform future research endeavors, and help introduce the analytic method to researchers and students. We discuss our findings in this section with these overarching aims, contrasting the findings from previous reviews in other areas of health care using visualization approaches.

### Gaps and Opportunities for Application Co-design

Data visualization aims to convey information *at a glance*, although it assumes that the audience has expertise and visual literacy on the subject matter [101]. In their review of visualization-based applications in infectious disease epidemiology, Carrol et al [14] summarize the audience’s information needs and learning behavior and point to 3 important barriers to relaying information to target audiences: (1) time constraints, (2) prior knowledge, and (3) cognitive load [14]. Hence, the design process is imperative for an effective application that allows the user to successfully understand the presented data. Various methodologies outlining effective design requirements and experiences from stakeholders to create new products and solutions have been explored [102-104]. For our scoping review, we opted to use the term *co-design*, which is more commonly used in health care literature, as opposed to *design thinking* and other related terms [13].

In this review, we found that at least two-thirds of the applications involved co-design approaches, involving stakeholders for developing interactive visualization applications. This was in contrast to a smaller proportion of co-designed VA applications (18%), which were mostly prototypes developed by and for data scientists at academic centers [13]. In line with this finding, more than half of the interactive visualization applications were developed in-house within health care organizations. We termed these initiatives *embedded research* as part of co-designed applications to indicate that these were initiated within the organizations in response to an identified data-driven need for knowledge generation and decision support. We could not find such applications in the VA literature [13]. This indicates an important trend because participatory design and development in health has proven to be a key element in better viability and uptake in planning and implementation of services [105,106].

Notably, a third of the articles in this review did not mention a co-design method, which could be due to authors either opting to omit it or because these were covered elsewhere. We recommend that future research indicate whether the application used co-design approaches. It is important to describe the context in adequate detail to appreciate stakeholder needs, experience, and satisfaction. Furthermore, to map and present methods in sufficient detail, we suggest using established frameworks such as the Munzner Nested Process [107] or Design Thinking for Visualization [108] as reporting tools.

### Contrasting Interactive Visualization and VA Applications

Through our recent work in studying visualization methods and applications in population health and HSR, we establish that the fields of interactive visualization and VA share communities of practice, methods, and approaches, but they are conceptually separate with important differences. We highlight the major ones here.

We found that interactive visualization applications were initiated by and targeted at researchers and practitioners within government health care organizations tasked with health services delivery, planning, and policy advice. In contrast, most of the VA applications were from and developed for data scientists [13]. In addition, most interactive visualization applications were developed using front-end engines, especially proprietary tools not requiring an advanced knowledge of coding [13]. Most VA methods and applications were prototypes developed using different combinations of tools, with a very small number using proprietary software [13]. Related to theoretical or conceptual frameworks, VA applications offered 13 different frameworks, whereas we could not identify any of these in this review of interactive visualization applications. However, the latter applications mentioned the use of frameworks at different stages of developing the applications. VA applications also expressly mentioned statistical and machine learning techniques as part of the analytic engine, whereas interactive visualization applications mostly used simple descriptive aggregative techniques. In another distinction, most VA applications were prototypes, whereas most interactive visualization applications were developed for knowledge generation and decision support [13].

Both the VA and interactive visualization techniques seem to have originated from North America and Europe [13]. The top 3 countries identified for VA applications were the United States (24/55, 44%), Canada (5/55, 9%), and Germany (3/55, 5%). The top countries for interactive visualization applications were the United States (30/56, 54%), the United Kingdom (4/56, 7%), and Canada and Indonesia (2/56, 4% each). Both our reviews indicated that most of the applications for both methods were descriptive analytics, with an overlap with exploratory analyses of complex data sets (23/55, 42% for VA and 37/56, 66% for interactive visualization), and a small proportion for predictive analytics. The application goals were comparable, with most being knowledge discovery (35/55, 80% for VA and 56/56, 100% for interactive visualization) or decision support (44/55, 80% for VA and 47/56, 84% for interactive visualization), with considerable overlap (29/55, 53% for VA and 47/56, 84% for interactive visualization). The data sets used for both types of applications were single (32/55, 58% for VA and 40/56, 71% for interactive visualization) and structured (40/55, 73% for VA and 48/56, 86% for interactive visualization). There were no unstructured data sets used for interactive visualization applications. Both types of applications used a small number of semistructured data sets (5/55, 9% for VA and 8/56, 14% for interactive visualization).

As population health and HSR are overlapping concepts, many articles in both reviews overlapped with their foci, methods, and the metrics studied. Among the VA articles, almost all (54/55, 98%) had a population health focus, whereas a third (18/55, 33%) were on HSR. There was a smaller overlap among the interactive visualization applications, with approximately two-thirds (38/56, 68%) focusing on population health and approximately half (29/56, 52%) on HSR.

Comparing the subdomains of population health and HSR, the 2 major categories of articles in the VA review focused on spatiotemporal aspects (27/55, 49%) compared with approximately a third (16/56, 29%) for interactive visualization applications. The next largest subdomains in VA included clinical populations focusing on a condition or cluster of conditions (17/55, 31%) or epidemic monitoring and modeling (18/55, 33%). Among the HSR articles for VA, these were mostly for health services’ utilization (15/55, 27%), access to care (10/55, 18%), or costs (2/55, 4%). Conversely, in the interactive visualization literature, the most common subdomain for population health was the study of a demographic population (28/56, 50%), followed by a clinical population (23/56, 41%), and epidemic monitoring and modeling (11/56, 20%). There was a similar trend toward the use of both interactive visualization in HSR, with the most common subdomains being health services’ utilization (23/56, 41%), followed by access (16/56, 29%) and costs (4/56, 7%).

The categories of problems have important similarities and variations with epidemiologic surveillance for infectious disease being the major category that the applications targeted (38% for both VA and interactive visualization). The next problem categories for VA applications were medical record pattern identification (20/55, 36%), population health monitoring (9/55, 16%), and health system resource planning (2/55, 4%). For interactive visualization applications, these included resources and services monitoring or planning (12/56, 21%), health service monitoring or quality (5/56, 9%), and medication use patterns (5/56, 9%).

Interactive visualization applications mostly used administrative and EMR or EHR data sources. This can be attributed directly to the availability of data within health care organizations. VA applications were developed using varied data sources, including administrative (19/55, 35%), EMR or EHR (17/55, 31%), spatiotemporal (16/55, 29%), social media (8/55, 15%), and simulation data (6/55, 11%); for interactive visualization applications, the data sources were secondary administrative data (45/56, 80%), social media (2/56, 4%), and sensor data (1/56, 2%).

Comparing tools in current use, about a third (21/55, 38%) of the VA applications were in use at the time of publication, whereas others were either not available or were prototypes. Moreover, a few (7/56, 13%) applications were accessible for public use, while less than a third were developed using free open source tools (13/56, 24%). Among the interactive visualization applications, more than half (31/56, 55%) were mentioned as being in current use, whereas about a third (18/56, 32%) were available to the public, and the same proportion were developed using free or open source tools. There was a greater proportion of use of proprietary tools (19/56, 34%) for interactive visualization applications compared only a 10th of VA applications (5/55, 10%).

The trend for the use of visual presentations was toward the use of different maps in both applications. Choropleth maps were the most frequently used for interactive visualizations (13/56, 24%), followed by thematic maps (10/56, 18%), event timelines (7/56, 13%), and network maps (4/56, 7%). VA applications showed a similar trend with thematic maps (17/55, 31%), timelines (8/55, 15%), and heat or choropleth maps (6/55, 11%). This corresponds to the findings of the review by Chung et al [17] on visualization methods in the area of mental health systems, which indicated that the most common means of presenting data was through maps [17].

Because of the differences in the methods involved in developing the applications, software tools varied greatly. VA tools were a mix of software tools used for the analytic and visual engines, whereas interactive visualization applications reported visual engines alone. However, there were still similarities in the use of tools. Tableau was the most frequently reported tool for interactive visualization applications (7/56, 13%), followed by D3.JS (5/56, 9%); ArcGIS and Instant Atlas (3/56, 5% each); and R/R-Shiny, Open Street Map, Google Maps API, SQL, and Java-based applications (2/56, 4% each). The most common tools found for VA applications were R-based tools (7/55, 13%), followed by D3.JS (4/55, 7%); SQL (4/55, 7%), Java-based tools (3/55, 5%); and Python-based tools, HTML 5, or Google Maps API (2/55, 4% each). Front-end visual engines such as Tableau were used by only 1 VA application in combination with Weka as the analytic engine.

Finally, an issue that we identified in both our reviews was the lack of reporting detail in the articles, which is important for the replicability and adaptation of the methods used in developing applications. We suggest using part of the VA Reporting Checklist that we presented in our previous work on VA, particularly around the details on the *visualization engine* for the standard reporting of interactive visualization applications [13].

### Recent Trends of Using Interactive Visualization Methods

Our results showed that thematic mapping, including choropleth maps, was the most common visual presentation across all problem categories of population health and HSR. This was particularly the case for epidemiologic monitoring and surveillance. The recently created COVID-19 dashboards fall into the same category of applications [109]. Mapping also surfaced as a popular method for health resource monitoring, particularly for the planning of health care services [41,56,62,64,67,73,77,78,80,87,110,111].

Among different conditions of interest, a significant number of applications were developed for studying trends in cancer [43,44,52,61,71,88]. Being a worldwide population health issue, the greater use of interactive visualization methods in cancer could be due to the availability of dedicated registries and secondary administrative data [88]. In global health, applications focused on surveillance of communicable diseases [47], outreach campaigns [64], methods to examine health inequalities [53], and effects on health from global climate change [61]. In HSR, 6 applications directly or indirectly highlighted inequities in health, particularly in regard to effective planning and advising policy [43,53,63,70,82,89]. There was 1 article examining social determinants of health in HIV [63].

As two-thirds of the applications were focused on the visual exploration of complex data sets, this indicated a clear trend toward the use of this technique for exploratory analyses. Although most applications were meant for descriptive analytics and visual exploration of complex data sets, of the 56 applications, 4 (7%) were also capable of predictive analytics [39,68,71,93]. The methodological frameworks that were applied to developing the applications pertained to visual monitoring [38], level of detail in visual presentation [80], use of scientific publication visualizations [45], information management [69], user-centered web-based applications [83], and user interaction [91].

### Opportunities for Future Applications and Research

Experts highlight the preference of researchers for interactive graphics to facilitate data exploration and abstraction, and they suggest greater, varied learning opportunities from the use of interactive visualization tools [14].

In comparison with standard, traditional statistical analyses, interactive visualization techniques can play an important complementary role through knowledge generation as well as establishing associations and causality. Interactive visualization methods enable a *data* discourse, leading to in-depth data-driven insights, while having the advantage of improved perception with reducing cognitive load [2,5]. This interplay of direct data manipulation and analysis allows simultaneous study of trends and patterns in the analytic process, while formulating and testing hypotheses [13,112]. Furthermore, these methods are considered apt for studying correlations in high-dimensional data with a large number of time points [112]. This translates into a powerful technique for using big health care data, allowing a deep exploratory dive without an a priori hypothesis to identify data-driven trends and patterns.

In this review, although we observed various applications of interactive visualization, we found limited evidence of its use in global health. Given the massive open access data sets available from agencies such as the World Bank and the World Health Organization (WHO), research can focus on studying a plethora of population health and HSR indicators [113,114]. The WHO’s Global Health Observatory provides population health–related data and statistics from 194 member states, particularly on nutrition, virological surveillance, workforce, and health systems, whereas the World Bank’s open data repository features macroeconomic and social indicators such as gender and aid effectiveness. The methods can be helpful in ecologic studies, such as those comparing indicators across and within nations.

Related to this is another major opportunity for the use of interactive visualization in studying inequities, especially those rooted in social determinants of health. Although the social determinants of health have become a major focus for investigating structural inequities, we found only 1 article examining related aspects in the HIV sector [63]. Social determinants of health are defined by the WHO as “conditions in which people are born, grow, live, work and age...shaped by the distribution of money, power and resources at global, national and local levels” [115]. This is especially relevant for investigating structural inequities related to issues of access and use based on race, gender, disability, income distribution, and indigenous populations [116,117]. Taking Canada’s example, investigating proximal factors for health among indigenous populations is one of the priority areas for improving health care [116]. Furthermore, high-quality Canadian data can be used to investigate inequities to better understand gaps in access and use of services by underserved populations. This can be done through national administrative data sources such as the Canadian National Ambulatory Care Reporting System, Discharge Abstract Database, and Hospital Morbidity Database, which store data for emergency and ambulatory care [118], as well as hospital inpatient discharges and day surgery [119].

Another major opportunity comes from the extension of using multiple data sources for studying patient journeys and care pathways. With the increasing use of EMR and EHR technologies, especially in primary care, there is an opportunity for researching patient populations along the continuum of care. Another such example is from the United Kingdom’s Clinical Practice Research Datalink database, which forms the largest collection of anonymized primary care patient records [120]. In Ontario, Canada, the Electronic Medical Record Administrative Data Linked Database offers high-quality linked data for exploring trends and patterns in care and its provision with the advantage of capturing quality of care measures involving prescriptions and investigations [121].

In a recent review on visualization approaches for supporting mental health systems and policy research, Chung et al [17] indicate that there is a gap in studies that influence policy. Although policy was not our main area of focus for this review, the work indicates that there is an opportunity for informing and advising policy based on the use of big data, especially in the important area of mental health services.

Although the potential for the use of interactive visualization tools for bringing together disparate data sources is valued, there are related concerns for data interpretation, quality, accuracy, and handling [1,14]. Meeting the needs of diverse users and interdisciplinary teams as well as promoting the understanding of visual approaches are 2 related and important challenges to be cognizant of [1,14]. Researchers indicate that understanding the value of these techniques among health care organizations and public health agencies is key to realizing the potential of these methods regarding decision support [17].

### Implications and Value-add From the Review

Our work is unique in several respects. Complementing our work on VA applications in population health and HSR [13], this review amalgamates the findings from studies on interactive visualization applications, while delineating the literature to construct a holistic picture on the use of visualization approaches in these areas of health care. Interactive visualization is an increasingly popular method, especially for embedded research within health care organizations. Although traditional statistical methods inform causality and associations of various conditions, interactive visualization presents a complementary opportunity for knowledge discovery, hypotheses generation, and decision support using big health care data.

As a novel method, we present findings from both our scoping reviews on VA and interactive visualization in a dynamic, interactive, and visual format using Tableau dashboards [37,122]. In the interest of greater transparency and replicability, we provide the abstraction database with relevant fields for adaptation and further analysis [37].

We highlight opportunities in areas of research that could benefit from visualization-based methods to promote the understanding and uptake of the methods among the communities of research and practice. This work would also prove useful in further developing visualization-related analytic methods.

### Limitations

Although there are several important limitations that we are cognizant of in reporting this review, we made extensive efforts to identify relevant literature, delineate the body of literature on interactive visualization applications, incorporated rigor in our methods through all stages, and went through extensive steps toward validation to present our findings.

We cast a wide net in our literature search covering 6 databases, published the study protocol, and had our search strategy externally peer reviewed. However, we may have missed relevant literature residing in subject-specific databases such as those of digital art, mathematics, geography, and computer science. In addition, our review was limited to peer-reviewed literature from journal articles and full conference papers, and we focused on health care–related databases. We did not include CINAHL and ACM Digital Library because we could not find unique articles, separate from MEDLINE and IEEE Xplore, during the pilot searches.

In addition, in line with the first review on VA, this literature synthesis is limited to articles published between January 1, 2005, and March 30, 2019. We situate and report the review within the same period as the one on VA applications to complement and contrast findings. Many COVID-19–related visualization products that surfaced later are not included in this review for both reasons of feasibility and the subject being extremely specialized and falling under *outbreak analytics*. However, we plan a rapid analysis of COVID-19–related visual products later in the year. Although we describe interactive visualization applications, we allude only briefly to the challenges in the use of these methods because this was beyond the scope of this review.

### Conclusions

Visualization in health has strong historical roots. This systematic literature synthesis informs the state of evidence and trends toward the use of interactive visualization methods in the important and interrelated areas of population health and HSR. We note a significant trend in the use of interactive visualization applications being used in health care organizations, which we term *embedded research*. Such applications are being used by academic and health care agencies for knowledge discovery and generation, as well as decision support. Many of these applications have been co-designed with relevant stakeholders. Although we found a wide array of applications in different subdomains of population health and health services, there are multiple opportunities for the use of these methods in investigating global- and national-level indicators and social determinants of health, as well as constructing patient journeys for a holistic picture of the continuum of care.

## Appendix

Multimedia Appendix 1 PRISMA-ScR (Preferred Reporting Items for Systematic Reviews and Meta-Analyses extension for Scoping Reviews) reporting checklist.

Multimedia Appendix 2 Details of problems analyzed, settings, and target audience.

Multimedia Appendix 3 Analytic capability and goals of the application.

Multimedia Appendix 4 Data types, sources, and visualization tools.

Multimedia Appendix 5 Functional types and details on visual presentations.

## Abbreviations

API: application programming interface
EHR: electronic health record
EMR: electronic medical record
GIS: geographic information system
HSR: health services research
PRISMA: Preferred Reporting Items for Systematic Reviews and Meta-Analyses
VA: visual analytics
WHO: World Health Organization
