# Supplementary material for: Interactive Visualization Applications in Population Health and Health Services Research: Systematic Scoping Review
Source: J Med Internet Res. 2022 Feb 18;24(2):e27534. doi: 10.2196/27534 (PMC8900899; doi:10.2196/27534)
Supplement: Multimedia Appendix 2 [file jmir_v24i2e27534_app2.docx]

# Multimedia Appendix 2: Problem analyzed, settings and target audience

| **Author and year** | **Country** | **Problem analyzed** | **SETTINGS** | | | **TARGET AUDIENCE** | | | | | |
| --- | --- | --- | --- | --- | --- | --- | --- | --- | --- | --- | --- |
|  |  |  | ***Government; ministry; health unit*** | ***Academic*** | ***Industry*** | ***Population or public health practitioners*** | ***Clinicians*** | ***Data scientists*** | ***Industry: software, pharmaceutical, insurance*** | ***Public and patient groups*** | ***Policy and decision makers*** |
| Alibrahim et al. 2014 [38] | USA | Developed a performance dashboard to assist in the monitoring of performance of more than 400 publicly funded treatment programs in Los Angeles. | x |  |  | x |  |  |  |  | x |
| Barrento et al. 2017 [39] | Portugal | Developed a dashboard to compare health benchmarks across European countries using benchmarking to see quality improvements, comparisons. and forecasts. | x |  |  | x |  |  |  | x |  |
| Basole et al. 2015 [40] | USA | Presented a multiple, coordinated visualization system to identify and analyze care processes and conformance to existing care guidelines using data of 5,784 pediatric emergency department visits over a 13-month period. | x |  |  |  | x |  |  |  |  |
| Becnel et al. 2019 [41] | USA | Designed an online interactive map reflecting distribution of Advanced Practice Registered Nurses, heightening their visibility and improving usability of data. |  | x |  | x | x |  |  |  | x |
| Benitez et al. 2017 [42] | Spain | Presented the design and construction of a web-based platform having an intuitive system with a graphical user interface eliminating the complexity behind questionnaire, data gathering, analysis and visualization. |  | x |  | x |  |  |  |  |  |
| Ben Ramadan et al. 2017 [43] | USA | Visualized database containing demographics, geospatial health data of female breast cancer incidence on a map of Missouri to inform researchers and policy makers about potential environmental and socioeconomic risk factors. | x |  |  | x | x | x |  |  |  |
| Ben Ramadan et al. 2018 [44] | USA | Developed tool to measure and interactively visualize survival data of female breast cancer cases in the Missouri Cancer Registry. | X |  |  | X | X |  |  |  |  |
| Bieh-Zimmert et al. 2013 [45] | Germany | Developed a scalable visualization technique focusing on a range of categorical information to analyze the existing data. |  | x |  | x |  |  |  | x |  |
| Bjarnadottir et al. 2016 [46] | USA | Applied EventFlow to analzyeanalyze patterns of prescription fills, gaps, and overlaps for pattern discovery, hypothesis generation, and study design. | x | x |  | x | x |  |  |  |  |
| Brownstein et al. 2010 [47] | USA | Designed a visualization system for continuous diseases data collection and processing for analysis of aggregate reports and geographic location. | x |  |  | x | x |  |  | x |  |
| Cesario et al. 2012 [48] | Brazil | Presented an investigation to use time-based visualization techniques with geographical maps and support for distributed mobile collection of patient data for disease surveillance. |  | x |  | x |  |  |  |  |  |
| Chui et al. 2011 [49] | USA | Proposed a graphical tool that can reveal the distribution of an outcome using multi-panel graphs on a number of data sets having different time periods, geographies, diseases. |  | x |  | x |  |  |  |  |  |
| Haque et al. 2014 [50] | Canada | Proposed Business business iIntelligence techniques application applied to health system infrastructure lessens tediousness and improves efficiency when examining legacy data. |  | x |  | x | x |  |  |  |  |
| Happe et al. 2018 [51] | France | Developed a tool box (ePEPS) for exploring patient cohort trajectories in a large dataset. |  | x |  | x | x |  |  |  |  |
| Henley et al. 2018 [52] | USA | Described CDC resources and visualizations that can be used to examine data on lung cancer incidence, survival, prevalence, and mortality among women. | x |  |  | x | x |  |  |  |  |
| Hosseinpoor et al. 2018 [53] | Indonesia | Presented Health Equity Assessment Toolkit (HEAT) Plus, a health inequality monitoring tool developed by WHO. | x |  |  | x |  |  |  | x |  |
| Hsu et al. 2018 [54] | USA | Presented the Environmental Health Channel, an interactive web-based tool for visualizing environmental sensing and public health data. |  | x |  | x |  |  |  | x | x |
| Iyer et al. 2017 [55] | USA | Descriped DataScope, a web-based tool for visually querying and exploring large datasets, and provided an overview of the design and architecture. |  | x |  | x |  |  |  |  |  |
| Jia et al. 2015 [56] | USA | Developed a web-based application that visualizes the CMS referral network focusing on physicians’ referrals to hospitals. | x |  |  | x | x |  |  | x | x |
| Kaushal et al. 2018 [57] | India | Visualized patient journeys using 3 different visualization techniques for individual and aggregate patterns. |  | x |  | x | x |  | x |  |  |
| Kirtland et al. 2014 [58] | USA | Described Diabetes Interactive Atlas, a web based application for viewing geographic patterns on diabetes in the USA. | x |  |  | x | x |  |  |  |  |
| Ko et al. 2018 [59] | Korea | Applied conventional regression analyses with data visualization for prescription patterns of antihypertensive drugs. | x |  |  |  | x |  |  |  |  |
| Krause et al. 2015 [60] | USA | Developed a centralized data repository and scalable informatics platform, with a data management and visualization solution. |  | x |  | x |  |  |  |  |  |
| Kubasek et al. 2013 [61] | Czech Republic | Provided overview of the SVOD web portal (System for Visualizing of Oncological Data) focused on population risk analyses related to cancer epidemiology and its integration with the FP7 project TaToo (Tagging Tool based on a Semantic Discovery Framework). | x |  |  | x |  |  |  | x |  |
| Lanzarone et al. 2017 [62] | Italy | Presented a visualization tool to support health planners in handling the data to better perform their re-planning activities for home-based care. | x |  |  |  | x |  |  |  | x |
| Lopez-DeFede et al. 2011 [63] | USA | Visualized existing data systems to examine STDs and HIV or AIDS diagnosis rates to explore potential county- level associations between HIV or AIDS diagnosis rates and socioeconomic disadvantage. | x |  |  | x |  |  |  |  |  |
| Mahler et al. 2015 [64] | Tanzania | Visualized MCHIP gathered data to identify the voluntary medical male circumcision needs. | x |  |  | x |  |  |  |  | x |
| Marshall et al. 2017 [65] | USA | Developed a collaborative, statewide online dashboard to provide the public with timely overdose surveillance data. | x |  |  | x | x |  |  |  | x |
| Martinez et al. 2016 [66] | USA | Visualized injury data for effective surveillance for injury prevention and control. |  | x |  | x |  |  |  |  |  |
| Mitrpanont et al. 2017 [67] | Malaysia | Evaluated the effectiveness of MedThaiVis as the new visualization tool for EHR data. | x |  |  | x | x |  |  |  |  |
| Moni et al. 2015 [68] | USA | Presented CytoCom, a platform based on Cytoscape, for network visualization and analysis of comorbidities. | x |  |  | x |  |  |  |  | x |
| Monsen et al. 2015 [69] | USA | Applied multiple visualizations using open source tools (Tableau, D3) to examine multiple databases to examine the quality of nursing care for nursing assessments, interventions, and service delivery outcomes. | x |  |  | x | x |  |  |  | x |
| Monsivais et al. 2018 [70] | UK | Presented Propensity to Cycle Tool (PCT) and the Food Environment Assessment Tool (Feat), two visualization tools in the UK, as case studies, drawing parallels and contrasts between them. | x |  |  | x |  |  |  |  | x |
| Mozumder et al. 2018 [71] | UK | Presented InterPreT as a visualization tool to facilitate communication of cancer stats to patients and the public. | x |  |  | x | x |  |  | x |  |
| Ortiz-Zuazaga et al. 2015 [72] | Puerto Rico | Presented SalHUD, a prototype web-based application for visualizing health data from Puerto Rico using interactive maps displaying years of potential life lost. |  | x |  | x |  |  |  | x |  |
| Pachauri et al. 2014 [73] | India | Applied visual analytics tools to enable the Ananya program managers and partners in decision making. | x |  |  | x |  |  |  |  |  |
| Palmer et al. 2019 [74] | UK | Applied visualization tool to explore the large and complex system of community care for patients aged 65 years and over, focussing on uses of multiple services and whether common patterns of referrals exist. | x |  |  | x | x |  |  |  |  |
| Pickle et al. 2010 [75] | USA | Presented spatial patterns of disease and local characteristics using micromaps. |  | x |  | x |  | x |  |  |  |
| Pike et al. 2017 [76] | Canada | Applied Atlas visualization tool to improve access to health care data and to tailor its display and interpretation. | x |  |  | x | x |  |  |  | x |
| Podgornik et al. 2007 [77] | USA | Visualized influenza data to improve tracking and facilitate public health officials to monitor and make decisions. | x |  |  | x |  |  |  |  | x |
| Pur et al. 2007 [78] | Slovenia | Developed a system for monitoring primary health care system visualizations to discover patterns, outliers and anomalies. | x |  |  | x |  |  |  |  | x |
| Raghupathi et al. 2018 [79] | USA | Analyzed state of chronic diseases in the USA, using data from the Centers for Disease Control and Prevention and applying visualization and descriptive analytics techniques. | x |  |  | x |  |  |  |  | x |
| Ratwani et al. 2015 [80] | USA | Developed two dashboards for MedStar Health using Tableau for Patient Safety Event Reporting System: Health facility level and system level.. | x |  | x | x | x |  |  |  |  |
| Rodriguez-Fernandez et al. 2016 [81] | Indonesia | Visualized the non-communicable disease-infectious disease overlap using EHR data. | x |  |  | x | x |  |  |  |  |
| Rowlingson et al. 2013 [82] | UK | Mapped incidence and prescription data for diabetes and ADHD using open source NHS data to show trends and meaningful variations. | x |  |  | x |  |  |  |  | x |
| Semple et al. 2013 [83] | USA | Presented an out- of- hospital cardiac arrest (OHCA) web mapping application to provide users with interactive maps, showing geographic pattern of cardiac arrest rates, bystander CPR rates, and survival rates. |  | x |  | x | x |  |  | x | x |
| Shen et al. 2018 [84] | USA | Visualized data to estimate vaccination coverage among the Medicare population using a geographic mapping tool displaying vaccination coverage among different geographic units. | x |  | x | x |  |  |  |  |  |
| Sims et al. 2011 [85] | USA | Visualized foodborne vibriosis data to identify new relationships and unique patterns of foodborne vibriosis in the United States. | x |  |  | x |  |  |  |  | x |
| Sopan et al. 2012 [86] | USA | Developed Community Health Map, a web-based tool, to visualize health performance, access, and quality indicators and demographics for facilitating policy maker decisions. | x |  |  | x |  |  |  |  | x |
| Toyoda et al. 2015 [87] | Japan | Visualized insurance claim data to support medical expenditure analysis for insurers or local government. | x |  |  | x |  |  | x |  | x |
| Tsoi et al. 2018 [88] | USA, UK, Costa Rica, Sweden, Croatia, Japan, Hong Kong and China | Applied visualization on WHO cancer registry data for over 20 years to interactively study global cancer distribution, line charts, demographic analysis to demonstrate historical cancer trends. |  | x |  | x |  | x |  | x |  |
| Valdiserri et al. 2018 [89] | USA | Presented case of AIDSVu, an interactive map of the U.SA. showing the data on HIV at national, state, and local levels using visualization. | x |  |  | x |  | x |  |  | x |
| van der Corput et al. 2014 [90] | Netherlands | Presented a new approach to visualize prescription data from four different perspectives: physician, patient, medicine, and prescription. | x |  |  | x | x |  |  | x |  |
| Wang et al. 2011 [91] | USA | Presented the usage data of Lifelines2 visualization system, and user comments collected over eight medical case studies. |  | x |  | x |  | x |  |  |  |
| Wang et al. 2018 [92] | USA | Analyzed the relationships between the prevalence of preterm birth, the biological mothers’ demographic and administrative dataset in areas of residence using visualization approach. | x |  |  | x |  |  |  |  | x |
| Zhang et al. 2011 [93] | China | Developed a public health and disease control web geographic information system to control and monitor the disease’s spread, | x |  |  | x |  |  |  |  | x |

**x = applicable category.**
