# Supplementary material for: Interactive Visualization Applications in Population Health and Health Services Research: Systematic Scoping Review
Source: J Med Internet Res. 2022 Feb 18;24(2):e27534. doi: 10.2196/27534 (PMC8900899; doi:10.2196/27534)
Supplement: Multimedia Appendix 3 [file jmir_v24i2e27534_app3.pdf]

#	Author and year	Analytic capability			Goal of the application/ method	
		Descriptive analytics	*Predictive analytics*	*Visual exploration of complex datasets*	*Knowledge discovery/ exploratory analysis*	*Decision support*
1	Alibrahim et al. 2014 [38]	x			x	x
2	Barrento et al. 2017 [39]	x	x		x	x
3	Basole et al. 2015 [40]	x			x	x
4	Becnel et al. 2019 [41]	x		x	x	x
5	Benitez et al. 2017 [42]	x		x	x	x
6	Ben Ramadan et al. 2017 [43]	x		x	x	x
7	Ben Ramadan et al. 2018 [44]	x		x	x	
8	Bieh-Zimmert et al. 2013 [45]	x		x	x	x
9	Bjarnadottir et al. 2016 [46]	x		x	x	x
10	Brownstein et al. 2010 [47]	x			x	
11	Cesario et al. 2012 [48]	x		x	x	
12	Chui et al. 2011 [49]	x		x	x	x
13	Haque et al. 2014 [50]	x			x	x
14	Happe et al. 2018 [51]	x		x	x	x
15	Henley et al. 2018 [52]	x			x	x
16	Hosseinpoor et al. 2018 [53]	x		x	x	x
17	Hsu et al. 2018 [54]	x		x	x	x
18	Iyer et al. 2017 [55]			x	x	
19	Jia et al. 2015 [56]	x			x	x

20	Kaushal et al. 2018 [57]	x		x	x	x
21	Kirtland et al. 2014 [58]	x			x	x
22	Ko et al. 2018 [59]	x			x	x
23	Krause et al. 2015 [60]	x		x	x	
24	Kubasek et al. 2013 [61]	x		x	x	x
25	Lanzarone et al. 2017 [62]	x		x	x	x
26	Lopez-DeFede et al. 2011 [63]	x		x	x	
27	Mahler et al. 2015 [64]	x		x	x	x
28	Marshall et al. 2017 [65]	x		x	x	x
29	Martinez et al. 2016 [66]	x		x	x	
30	Mitrpanont et al. 2017 [67]	x			x	x
31	Moni et al. 2015 [68]	x	x	x	x	x
32	Monsen et al. 2015 [69]	x			x	x
33	Monsivais et al. 2018 [70]	x			x	x
34	Mozumder et al. 2018 [71]		x		x	
35	Ortiz-Zuazaga et al. 2015 [72]	x		x	x	x
36	Pachauri et al. 2014 [73]	x		x	x	x
37	Palmer et al. 2019 [74]	x		x	x	x
38	Pickle et al. 2010 [75]	x		x	x	x
39	Pike et al. 2017 [76]	x		x	x	x
40	Podgornik et al. 2007 [77]	x		x	x	x
41	Pur et al. 2007 [78]	x		x	x	x

42	Raghupathi et al. 2018 [79]	x			x	
43	Ratwani et al. 2015 [80]	x		x	x	x
44	Rodriguez-Fernandez et al. 2016 [81]	x		x	x	x
45	Rowlingson et al. 2013 [82]	x		x	x	x
46	Semple et al. 2013 [83]	x		x	x	x
47	Shen et al. 2018 [84]	x			x	x
48	Sims et al. 2011 [85]			x	x	x
49	Sopan et al. 2012 [86]	x		x	x	x
50	Toyoda et al. 2015 [87]	x			x	x
51	Tsoi et al. 2018 [88]	x		x	x	x
52	Valdiserri et al. 2018 [89]	x		x	x	x
53	van der Corput et al. 2014 [90]	x		x	x	x
54	Wang et al. 2011 [91]	x		x	x	x
55	Wang et al. 2018 [92]	x		x	x	x
56	Zhang et al. 2011 [93]	x	x		x	x
	**x=applicable category**					
